# Insulin-Induced Electrophysiology Changes in Human Pleura Are Mediated via Its Receptor

**DOI:** 10.1155/2010/853176

**Published:** 2010-08-12

**Authors:** V. K. Kouritas, M. Ioannou, C. N. Foroulis, N. Desimonas, K. Evaggelopoulos, K. I. Gourgoulianis, P. A. Molyvdas, C. Hatzoglou

**Affiliations:** ^1^Department of Physiology, Medical School, University of Thessaly, Mezourlo, P.O. Box 1400, New Buildings, 41100 Larissa, Greece; ^2^Department of Histopathology, Larissa University Hospital, 411 10 Larissa, Greece; ^3^Department of Cardiothoracic Surgery, Larissa University Hospital, 411 10 Larissa, Greece; ^4^Department of Thoracic Diseases, Larissa University Hospital, 411 10 Larissa, Greece

## Abstract

*Background*. Insulin directly changes the sheep pleural electrophysiology. The aim of this study was to investigate whether insulin induces similar effects in human pleura, to clarify insulin receptor's involvement, and to demonstrate if glibenclamide (hypoglycemic agent) reverses this effect. *Methods*. Human parietal pleural specimens were mounted in Ussing chambers. Solutions containing insulin or glibenclamide and insulin with anti-insulin antibody, anti-insulin receptor antibody, and glibenclamide were used. The transmesothelial resistance (*R*_TM_) was determined. Immunohistochemistry for the presence of Insulin Receptors (IRa, IRb) was also performed. *Results*. Insulin increased *R*_TM_ within 1st min (*P* = .016), when added mesothelially which was inhibited by the anti-insulin and anti-insulin receptor antibodies. Glibenclamide also eliminated the insulin-induced changes. Immunohistochemistry verified the presence of IRa and IRb. *Conclusion*. Insulin induces electrochemical changes in humans as in sheep via interaction with its receptor. This effect is abolished by glibenclamide.

## 1. Introduction

Insulin is one of the chief mediators of anabolism and glucose controllers. Diabetic patients who receive insulin as treatment may develop a rare but potentially dangerous complication known as “the insulin oedema syndrome” which is characterized from oedema and fluid formation in various sites of the body, ranging from simple ankle oedema to heavy cardiopulmonary manifestations, such as pulmonary oedema, cardiogenic shock and pleural effusions. [1–4]. 

A possible explanation for the insulin oedema syndrome was proposed to involve the ability of insulin to induce permeability alterations in epithelia such as the distal kidneys, at epithelial cell level, where it induces electrolyte and water retention [5–7] leading to fluid excess in the organism. 

However, despite the aforementioned explanation, the formation of pleural effusions in diabetic patients who develop the insulin oedema syndrome remains unexplained. In an effort to explain this event, a direct effect of insulin in pleura was previously demonstrated in sheep [8]; insulin induced electrochemical changes increasing the pleural trans-mesothelial resistance (*R*_TM_) by blocking ion transporters such as the amiloride-sensitive Na^+^ channels and the ouabain-sensitive Na^+^/K^+^ pumps who have been implicated in pleural fluid recycling [9]. In that study, the insulin receptors a (IRa) and b (IRb) were demonstrated to be present in sheep pleura, and therefore the aforementioned effect was suggested to be mediated by a possible insulin-insulin receptor interaction [8]. 

The aim of this study was to investigate if insulin effect on the electrochemical profile of the human parietal pleura follows similar pattern as in sheep, to clarify if an interaction with its receptor is involved, and to additionally investigate if glibenclamide (another hypoglycemic agent also used for diabetes treatment) can reverse this effect.

## 2. Materials and Methods

### 2.1. Human Parietal Pleural Specimens

Intact sheets of human parietal pleura were obtained from forty-four (44) patients, who underwent thoracic surgery for lung cancer (via thoracotomy or thoracoscopic procedures) for diagnostic and/or therapeutic purposes. The lung mass was not in proximity with the dissected specimens. A piece from each specimen was sent for histopathological examination, and all specimens used in the study were proven to be free of any disease, as per the histopathology report. Patients with pleural effusion prior to the operation, abnormal bloodstream glucose level preoperatively, or history of diabetes were excluded from the study. The remaining specimens were then placed in preoxygenated (bubbled with 95% O_2_—5% CO_2_) Krebs solution, cooled at 4°C, and were transferred to the laboratory within 30 minutes from tissue dissection. 

The study was approved by the Local Ethics Committee (Institutional Review Board), and signed consent was obtained from all the participated in the study patients.

### 2.2. Electrophysiology Studies

The KRB solution used throughout the whole study was balanced at pH 7.45 and contained 117.5 mM NaCl, 1.15 mM NaH_2_PO_4_, 24.99 mM NaHCO_3_, 5.65 mM KCl, 1.18 mM MgSO_4_, 2.52 mM CaCl_2_ and 5.55 mM Glucose. 

The surface of the pleura that faces in vivo the pleural cavity, will be referred to as the *mesothelial surface*, and the surface that faces the chest wall will be referred to as the *interstitial surface*.

The pleural tissues were mounted as planar sheets of tissue in *Ussing*-*type chambers* [8–10]. The tissue was bathed in Krebs solution on both sides and bubbled continuously with 95% O_2_—5% CO_2_ gas mixture, heated to 37°C, in order to ensure tissue viability. 

Following the equilibration period and control measurements [8, 10], insulin (bovine pancreas insulin, Sigma Chemical Co., USA) solutions (10^−7^ M) were added on the mesothelial and interstitial surface of the specimens (*n* = 7 experiments for each side) [8]. In other experiments the anti-insulin antibody (Sigma Chemical Co., USA, 50 mg/dl, dilution 1 : 80000) was added in insulin solutions (*n* = 7 experiments). Similarly, the anti-insulin receptor antibody (IR, pTyr^972^, Sigma Chemical Co., USA) or the anti-insulin-like growth factor 1 receptor antibody (Sigma Chemical Co., USA) was also added in other insulin solutions (*n* = 7 experiments). In other experiments glibenclamide (10^−5^ M) (Sigma Chemical Co., USA) was added on the mesothelial and interstitial surface of the specimens (*n* = 7 experiments for each side of the tissue). Finally, insulin 10^−7^ M was added in specimens (*n* = 7) pretreated with KRB solution with glibenclamide 10^−5^ M for at least 30 minutes prior to the experiments. 

PD_TM_ was measured 1, 5, 10 and 30 minutes after each solution addition and *Trans-mesothelial Resistance* (*R*_TM_) was calculated from PD_TM_ [8–10].

### 2.3. Statistical Analysis

Statistical analysis was performed using the statistical package SPSS ver. 10.00 for Windows (Statistical Package for the Social Sciences, SPSS Inc., Chicago, Ill., USA). Data are expressed as Mean *R*_TM_ (Ω·cm^2^) ± Standard Error of Mean (S.E.). Statistical significance between pairs was determined by student's paired *t*-test whereas between multiple groups by ANOVA (Bonferoni's post hoc). *P* values less than  .05 were considered significant.

### 2.4. Immunohistochemistry

Tissue sections (3 *μ*) from parietal human pleura were dried onto slights overnight at 60°. After deparaffinization in xylene and rehydration in decreasing ethanol solutions, slides were heated in target retrieval solution (pH 9, DAKO Denmark) for 25 minutes in a microwave oven (LG WAVEDOM 850 Watt). The sections were cooled, washed in PBS, and incubated in 0.3% hydrogen peroxide for 10 minutes to block endogenous peroxidase. After washing with PBS, 75 *μ*L of blocking buffer (DAKO, Carpinteria CA, USA) was added to each section for 1 hour. Then, the sections were incubated with the primary antibody to Insulin receptor a (rabbit polyclonal Santa Cruz Biotechnology, Santa Cruz CA) and Insulin receptor b, (rabbit polyclonal Santa Cruz Biotechnology, Santa Cruz CA) at 1 : 300 and 1 : 200 dilution, respectively, for 1 hour in room temperature. After the incubation, envision fluid (polymer-peroxidase method, EnVision+/HRP, DAKO Denmark) was added, followed by incubation for 30 minutes. The slides were counterstained with hematoxylin and mounted. Tissue sections from pancreas and liver were used as positive controls. Blood vessels wall in the mesothelium specimens were considered also as positive controls. For the negative control the incubation step with the primary antibody was omitted.

## 3. Results

### 3.1. Effect of Insulin in Human Parietal Pleura

**Figure 1:**
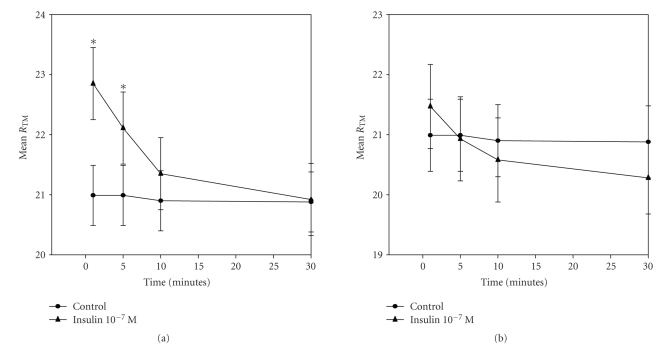
Effect of insulin 10^−7^ M addition on the Trans-mesothelial Resistance (*R*_TM_) when added on the mesothelial (a) and interstitial (b) surface of human parietal pleura, by time. Values are expressed as Mean Trans-mesothelial Resistance (Ω·cm^2^) ± Standard Error of Mean; *n* = 7 experiments. *P < .05 versus control.

Addition of insulin on the mesothelial surface increased *R*_TM_ rapidly, within the 1st minute (from 20.99 ± 0.5 Ω·cm^2^ to 22.85 ± 0.6 Ω·cm^2^, dR_TM_ 1.86 Ω·cm^2^, versus control *P* = .016). This effect lasted for 5 min (22.11 ± 0.6 Ω·cm^2^, versus control, *P* = .026), and *R*_TM_ was decreased thereafter till baseline (20.92 ± 0.6 Ω·cm^2^, versus control, *P* > .05) after 30 minutes (Figure 1(a)). Little effect was observed interstitially (20.99 ± 0.5 Ω·cm^2^ to 21.47 ± 0.6 Ω·cm^2^, dR_TM_ 0.48 Ω·cm^2^, versus control *P* > .05) (Figure 1(b)).

### 3.2. Effect of Anti-Insulin Antibody on Insulin-Induced Alterations

**Figure 2:**
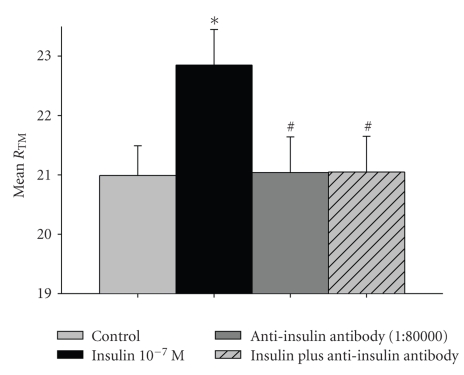
Effect of Anti-Insulin antibody on the insulin-induced electrochemical alterations in human parietal pleura. Values are expressed as Mean of Trans-mesothelial Resistance (Ω·cm^2^) ± Standard Error of Mean of *n* = 7 for each set of experiments. *P < .05  versus control, ^#^ *P* < .05 versus insulin.

The anti-insulin antibody totally inhibited the insulin-induced effect (from 22.85 ± 0.6 Ω·cm^2^ to 21.05 ± 0.6 Ω·cm^2^ *P* = .01, versus control *P* > .05, Figure 2).

### 3.3. Effect of Anti-Insulin Receptor Antibody on Insulin-Induced Alterations

**Figure 3:**
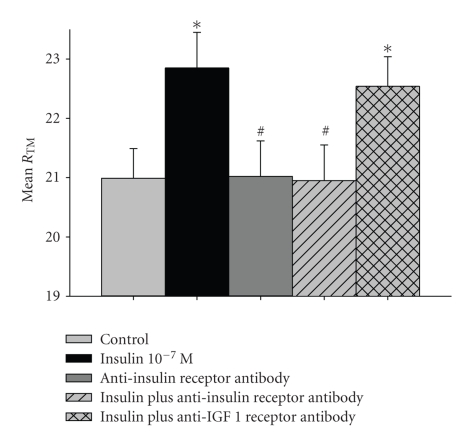
Effect of Anti-Insulin Receptor antibody and Anti-IGF 1 Receptor antibody on the insulin-induced electrochemical alterations in human parietal pleura. Values are expressed as Mean of Trans-mesothelial Resistance (Ω·cm^2^) ± Standard Error of Mean of *n* = 7 for each set of experiments. *P < .05 versus control, ^#^ *P* < .05 versus insulin.

The anti-insulin receptor antagonist also totally inhibited insulin-induced effect (from 22.85 ± 0.6 Ω·cm^2^ to 20.95 ± 0.6 Ω·cm^2^ *P* = .01, versus control *P* > .05, Figure 3).

### 3.4. Effect of Anti-Insulin-Like Growth Factor 1 (IGF) Receptor Antibody on Insulin-Induced Alterations

The anti-IGF-1 receptor antagonist did not inhibit the insulin-induced effect (from 22.85 ± 0.6 Ω·cm^2^ to 22.54 ± 0.5 Ω·cm^2^ *P* > .05, versus control *P* = .014, Figure 3).

### 3.5. Effect of Glibenclamide in Human Parietal Pleura and on Insulin-Induced Alterations

**Figure 4:**
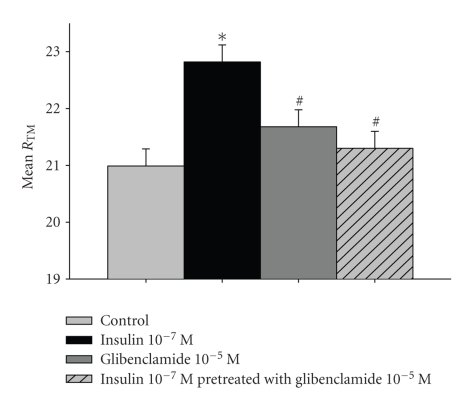
Effect of Glibenclamide 10^−5^ M on the insulin-induced electrochemical alterations in human parietal pleura. Values are expressed as Mean of Trans-mesothelial Resistance (Ω·cm^2^) ± Standard Error of Mean of *n* = 7 for each set of experiments. *P < .05 versus control, ^#^ *P* < .05 versus insulin.

Addition of glibenclamide had no effect on *R*_TM_ either when added mesothelially (weak increase of 0.54 Ω·cm^2^, versus control *P* > .05, Figure 4) or interstitially. *R*_TM_ remained near baseline throughout the experiments. Glibenclamide abolished the insulin-induced (Figure 4) *R*_TM_ increase from the 1st min of coaddition (from 20.99 ± 0.6 Ω·cm^2^ to 21.30 ± 0.5 Ω·cm^2^, dR_TM_ 0.31 Ω·cm^2^, versus insulin *P* = .022, versus control *P* > .05).

### 3.6. Detection of IRa and IRb in Human Parietal Pleura

**Figure 5:**
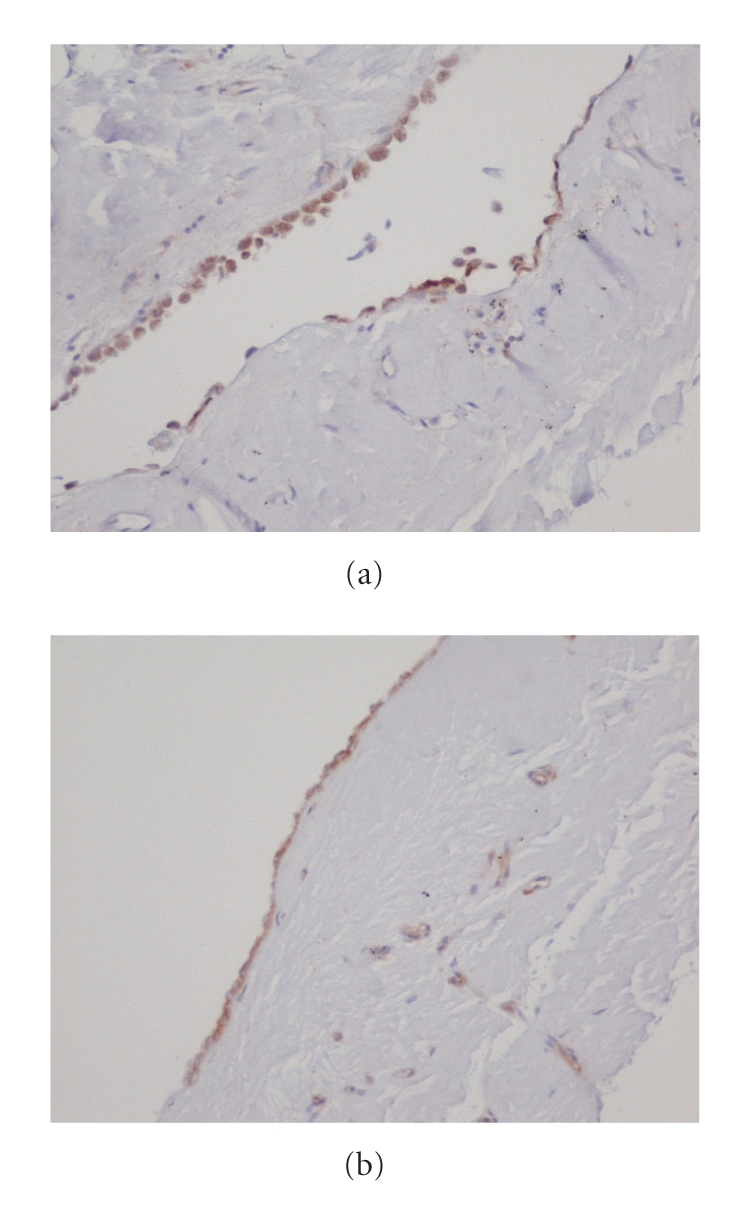
(a) IRa cytoplasmic immunoreactivity in mesothelial cells of parietal mesothelial tissue (immunoperoxidase stain, original magnification 2 × 20). (b) IRb positivity in mesothelial cells of parietal mesothelial tissue (immunoperoxidase stain, original magnification 2 × 20).

Mesothelial cells showed positive immunostaining for IRa and IRb. The immunoreactivity was cytoplasmic (Figures 5(a) and 5(b)). The distribution of immunoreactivity was diffuse. Staining intensity was even and convincing.

## 4. Discussion

The main finding of this study is that insulin induced electrochemical changes in human parietal pleura when added on the mesothelial surface. This effect is elicited after interaction of insulin with its receptors which were identified as per the immunohistochemistry to be present in human parietal pleura. This insulin-induced effect is reversed by another commonly used hypoglycaemic agent, glibenclamide, which had a weak effect on the electrochemical profile of the human parietal pleura.

Insulin induces similar effects in other tissues such as the toad urinary bladder where insulin increased short circuit current within the first 5 minutes towards the mesothelial side of tissue [11]. Insulin showed an increase of PD_TM_ of alveolar type II cells when added mesothelially [12, 13]. Interstitial effect of insulin due to diffusion was cited in toad urinary bladder [11]. Weak interstitial effect was observed in this study possibly due to diffusion [14, 15] or because of remnants of fat tissue or blood clots [16]. In kidney cells insulin is known to stimulate amiloride-sensitive Na^+^ channels when added apically [6, 7, 11]. 

In the present study, insulin was used in a concentration of 10^−7^ M, given that this concentration was previously shown to be the least effective concentration in sheep [17]. In insulin-treated humans, insulin levels rarely reach such high concentrations (i.e., in poorly controlled patients), suggesting that the pleura can be directly stimulated by insulin only with high concentrations, explaining in this way the rarity of pleural effusions during insulin therapy [2]. Insulin is possibly diffused into the pleural cavity according to bloodstream levels following diabetes treatment. This high concentration could also explain the fact that in pleura insulin decreases the permeability rather than augments it, as is the case in kidneys. High insulin concentrations can produce different effect patterns in different epithelia or even in the epithelia of the same target organs that is, the kidneys were even interstitial effect was recorded when insulin concentrations used were high [18]. 

It has been commonly demonstrated that insulin augments glucose uptake via interaction with its receptor. Additionally, insulin interferes with Na^+^ transportation in epithelial tissues via interaction with its receptor [6, 7, 19, 20]. Similar permeability regulation by insulin via a receptor-mediated process was shown in T84 colonic cells [21]. Insulin receptors were demonstrated to be more abundant on the basolateral side of human bronchial epithelial cells [22]. Results from the present study show that insulin and its interaction with its receptors a (IRa) and b (IRb) induce the observed electrochemical alterations in the human parietal pleura. 

From all the aforementioned, insulin induced a comparable electrochemical effect in sheep and human pleura. Additionally, the insulin receptors a (IRa) and b (IRb) were also shown to exist in both species. Therefore, electrophysiological and histopathological observations present many similarities in sheep and human pleura suggesting that sheep can be an acceptable animal model for observations that may be extrapolated to humans. This finding is important given that the human tissue is hard to be obtained due to the fact that consent is needed the healthy subjects are not subjected to surgery whereas its stripping leads to bleeding [8]. 

IGF-1 also produces transcellular ion fluxes across epithelial tissues and has been implicated to change the permeability that is, of the kidneys [23, 24] after interaction with its receptor. IGF-1 and insulin receptors have similar structure, and insulin may interact with IGF-1 receptor. If insulin induces its effect by binding to the IGF-1 receptor, then the inhibition of the insulin receptor would not have inhibited the insulin's effect in pleura, given that the IGF-1 receptor would have been free to bind with insulin. If insulin induces its effect by binding to the insulin/IGF-1 receptor, then the inhibition of the insulin receptor would have partially inhibited the insulin's effect in pleura. The results of the coaddition of insulin with the anti-IGF-1 receptor antibody in some specimens suggest that the IGF-1 receptor is not involved with the insulin effect in pleura at this at least concentration (10^−7^ M). However, more research is warranted in order to clarify the involvement of the IGF-1 receptor in the insulin effect in pleura.

Glibenclamide is an antidiabetic agent which enhances insulin production by *β*-pancreatic cells via inhibition of adenosine triphosphate sensitive K^+^ channels (K(ATP)) [25, 26]. Such channels are present in kidneys [27, 28] constituting glibenclamide as an agent interfering with the electrolyte transportation [29–31]. However, its involvement in the function of mitochondrial K(ATP) attributed to this drug a possible harmful effect [32]. Such harmful effect in terms of electrophysiology was not supported by our results. More importantly, glibenclamide inhibited the insulin-induced electrochemical effect in pleura. This inhibition is explainable as it depletes the cellular ATP content, and therefore the insulin-insulin receptor binding may be hindered. The blockage of K^+^ transportation via K(ATP) channels by glibenclamide needs further clarification in pleura since its addition did not induce electrochemical and permeability changes suggesting that such channels do not exist in pleura. Apart from its hypoglycemic role, glibenclamide was additionally chosen for its effects in kidneys and myocardium [33, 34].

Results from the present study indicate that theoretically insulin presence in the pleural cavity makes the pleural membrane less permeable [8, 35], and this event may provide an additional explanation for the formation of pleural effusion in insulin-treated diabetic patients. Insulin may therefore present oedematic properties by altering the electrophysiological profile and consequently interfere with pleural recycling [35]. This oedematic effect can be reversed by another hypoglycaemic agent such as glibenclamide. This observation, although at experimental level, may provide an alternative treatment option for clinicians who encounter this rare complication of pleural effusion formation during insulin therapy. 

## 5. Conclusions

In conclusion, insulin induced electrophysiology alterations of the human parietal pleura which were similar with the effect produced in sheep pleura and is mediated by interacting with its receptor. This finding alongside with the fact that these receptors were demonstrated to be present in human parietal pleura as in sheep implicates the similarity of the parietal pleura of the two species. The hypoglycaemic agent glibenclamide totally inhibited this effect without inducing intense electrophysiological alterations of the human parietal pleura.

## References

[1] Chelliah A, Burge MR (2004). Insulin edema in the twenty-first century: review of the existing literature. *Journal of Investigative Medicine*.

[2] Kalambokis GN, Tsatsoulis AA, Tsianos EV (2004). The edematogenic properties of insulin. *American Journal of Kidney Diseases*.

[3] Lee P, Kinsella J, Borkman M, Carter J (2007). Bilateral pleural effusions, ascites, and facial and peripheral oedema in a 19-year-old woman 2 weeks following commencement of insulin lispro and detemir—an unusual presentation of insulin oedema. *Diabetic Medicine*.

[4] Zenda T, Murase Y, Yoshida I, Muramoto H, Okada T, Yagi K (2003). Does the use of insulin in a patient with liver dysfunction increase water retention in the body, i.e. cause insulin oedema?. *European Journal of Gastroenterology and Hepatology*.

[5] Nofziger C, Chen L, Shane MA, Smith CD, Brown KK, Blazer-Yost BL (2005). PPAR*γ* agonists do not directly enhance basal or insulin-stimulated Na^+^ transport via the epithelial Na^+^ channel. *Pflügers Archiv European Journal of Physiology*.

[6] Blazer-Yost BL, Cox M, Furlanetto R (1989). Insulin and IGF I receptor-mediated Na^+^ transport in toad urinary bladders. *American Journal of Physiology*.

[7] Blazer-Yost BL, Liu X, Helman SI (1998). Hormonal regulation of eNaCs: insulin and aldosterone. *American Journal of Physiology*.

[8] Hatzoglou CH, Gourgoulianis KI, Molyvdas PA (2001). Effects of SNP, ouabain, and amiloride on electrical potential profile of isolated sheep pleura. *Journal of Applied Physiology*.

[9] Kouritas VK, Hatzoglou C, Ioannou M, Gourgoulianis KI, Molyvdas PA (2010). Insulin alters the permeability of sheep pleura. *Experimental and Clinical Endocrinology and Diabetes*.

[10] Kouritas VK, Hatzoglou C, Foroulis CN, Hevas A, Gourgoulianis KI, Molyvdas PA (2007). Low glucose level and low pH alter the electrochemical function of human parietal pleura. *European Respiratory Journal*.

[11] Cox M, Singer I (1977). Insulin mediated Na^+^ transport in the toad urinary bladder. *American Journal of Physiology*.

[12] Sugahara K, Freidenberg GR, Mason RJ (1984). Insulin binding and effects on glucose and transepithelial transport by alveolar type II cells. *American Journal of Physiology*.

[13] Marunaka Y, Niisato N, O’Brodovich H, Post M, Tanswell AK (1999). Roles of Ca^2+^ and protein tyrosine kinase in insulin action on cell volume via Na^+^ and K^+^ channels and Na^+^/K^+^/2Cl^^−^^ cotransporter in fetal rat alveolar type II pneumocyte. *Journal of Membrane Biology*.

[14] Yamamoto A, Tanaka H, Okumura S (2001). Evaluation of insulin permeability and effects of absorption enhancers on its permeability by an in vitro pulmonary epithelial system using Xenopus pulmonary membrane. *Biological and Pharmaceutical Bulletin*.

[15] Carstens S, Danielsen G, Guldhammer B, Frederiksen O (1993). Transport of insulin across rabbit nasal mucosa in vitro induced by didecanoyl-L-*α*-phosphatidylcholine. *Diabetes*.

[16] Ribière C, Jaubert A-M, Sabourault D, Lacasa D, Giudicelli Y (2002). Insulin stimulates nitric oxide production in rat adipocytes. *Biochemical and Biophysical Research Communications*.

[17] Kosior-Korzecka U, Bobowiec R, Lipecka CZ (2006). Fasting-induced changes in ovulation rate, plasma leptin, gonadotropins, GH, IGF-I and insulin concentrations during oestrus in ewes. *Journal of Veterinary Medicine Series A*.

[18] Herrera FC (1965). Effect of insulin on short-circuit current and sodium transport across toad urinary bladder. *American Journal of Physiology*.

[19] Hammerman MR (1985). Interaction of insulin with the renal proximal tubular cell. *American Journal of Physiology*.

[20] Deachapunya C, Palmer-Densmore M, O’Grady SM (1999). Insulin stimulates transepithelial sodium transport by activation of a protein phosphatase that increases Na-K ATPase activity in endometrial epithelial cells. *Journal of General Physiology*.

[21] McRoberts JA, Riley NE (1992). Regulation of T84 cell monolayer permeability by insulin-like growth factors. *American Journal of Physiology*.

[22] Pezron I, Mitra R, Pal D, Mitra AK (2002). Insulin aggregation and asymmetric transport across human bronchial epithelial cell monolayers (Calu-3). *Journal of Pharmaceutical Sciences*.

[23] Blazer-Yost BL, Record RD, Oberleithner H (1996). Characterization of hormone-stimulated Na^+^ transport in a high resistance clone of the MDCK cell line. *Pflügers Archiv European Journal of Physiology*.

[24] Staruschenko A, Pochynyuk O, Vandewalle A, Bugaj V, Stockand JD (2007). Acute regulation of the epithelial Na^+^ channel by phosphatidylinositide 3-OH kinase signaling in native collecting duct principal cells. *Journal of the American Society of Nephrology*.

[25] Panten U, Schwanstecher M, Schwanstecher C (1996). Sulfonylurea receptors and mechanism of sulfonylurea action. *Experimental and Clinical Endocrinology and Diabetes*.

[26] Aguilar-Bryan L, Bryan J (1999). Molecular biology of adenosine triphosphate-sensitive potassium channels. *Endocrine Reviews*.

[27] Quast U (1996). ATP-sensitive K^+^ channels in the kidney. *Naunyn-Schmiedeberg’s Archives of Pharmacology*.

[28] Engbersen R, Moons MM, Wouterse AC (2000). Sulphonylurea drugs reduce hypoxic damage in the isolated perfused rat kidney. *British Journal of Pharmacology*.

[29] Huang DY, Osswald H, Vallon V (2000). Sodium reabsorption in thick ascending limb of Henle’s loop: effect of potassium channel blockade in vivo. *British Journal of Pharmacology*.

[30] Wang T (2003). The effects of the potassium channel opener minoxidil on renal electrolytes transport in the loop of Henle. *Journal of Pharmacology and Experimental Therapeutics*.

[31] Gosmanov AR, Fan Z, Mi X, Schneider EG, Thomason DB (2004). ATP-sensitive potassium channels mediate hyperosmotic stimulation of NKCC in slow-twitch muscle. *American Journal of Physiology*.

[32] Engbersen R, Masereeuw R, van Gestel MA, van der Logt EMJ, Smits P, Russel FGM (2005). Glibenclamide depletes ATP in renal proximal tubular cells by interfering with mitochondrial metabolism. *British Journal of Pharmacology*.

[33] Clark MA, Humphrey SJ, Smith MP, Ludens JH (1993). Unique natriuretic properties of the ATP-sensitive K^+^-channel blocker glyburide in conscious rats. *Journal of Pharmacology and Experimental Therapeutics*.

[34] Yellon DM, Downey JM (2003). Preconditioning the myocardium: from cellular physiology to clinical cardiology. *Physiological Reviews*.

[35] Lai-Fook SJ (2004). Pleural mechanics and fluid exchange. *Physiological Reviews*.

